# Bone marrow-derived mesenchymal stem cells combined with gonadotropin therapy restore postnatal oogenesis of chemo-ablated ovaries in rats via enhancing very small embryonic-like stem cells

**DOI:** 10.1186/s13287-021-02415-5

**Published:** 2021-09-27

**Authors:** Nesrine Ebrahim, Hajir A. Al Saihati, Amani Shaman, Arigue A. Dessouky, Ayman Samir Farid, Noha I. Hussien, Ola Mostafa, Yasmin Seleem, Dina Sabry, Ahmed S. Saad, Hanan Tawfeek Emam, Amira Hassouna, Omnia A. M. Badr, Bayan A. Saffaf, Nicholas R. Forsyth, Rabab F. Salim

**Affiliations:** 1 Department of Histology and Cell Biology, Faculty of Medicine, Benha University, Benha, Egypt; 2 Stem Cell Unit, Faculty of Medicine, Benha University, Benha, Egypt; 3 Department of Clinical Laboratory Sciences, College of Applied Medical Sciences, University of Hafr Albatin, Al-Batin, Saudi Arabia; 4 Obstetrics and Gynecology Medical College, Tabuk University, Tabuk, Saudi Arabia; 5 Department of Medical Histology and Cell Biology, Faculty of Medicine, Zagazig University, Zagazig, Egypt; 6 Department of Clinical Pathology, Faculty of Veterinary Medicine, Benha University, Benha, Egypt; 7 Department of Medical Physiology, Faculty of Medicine, Benha University, Benha, Egypt; 8 Department of Clinical Pharmacology, Faculty of Medicine, Benha University, Benha, Egypt; 9 Department of Medical Biochemistry and Molecular Biology, Faculty of Medicine, Badr University, Cairo, Egypt; 10 Department of Obstetrics & Gynecology, Faculty of Medicine, Benha University, Benha, Egypt; 11 School of Interprofessional Health Studies, Faculty of Health and Environmental Sciences, AUT University, Auckland, New Zealand; 12 Department of Genetics and Genetic Engineering, Faculty of Agriculture, Benha University, Benha, Egypt; 13 Department of pharmacology, Faculty of Pharmacy, Future University, Cairo, Egypt; 14 Guy Hilton Research Laboratories, School of Pharmacy and Bioengineering, Faculty of Medicine and Health Sciences, Keele University, Keele, UK; 15 Department of Medical Biochemistry and Molecular Biology, Faculty of Medicine, Benha University, Benha, Qalyubia 13512 Egypt

**Keywords:** Ovarian chemo-ablation, MSCs, Postnatal oogenesis, VSELs, Gonadotropins

## Abstract

**Background:**

Very small embryonic-like stem cells (VSELs) are a rare population within the ovarian epithelial surface. They contribute to postnatal oogenesis as they have the ability to generate immature oocytes and resist the chemotherapy. These cells express markers of pluripotent embryonic and primordial germ cells.

**Objective:**

We aimed to explore the capability of VSELs in restoring the postnatal oogenesis of chemo-ablated rat ovaries treated with bone marrow-derived mesenchymal stem cells (BM-MSCs) combined with pregnant mare serum gonadotropin (PMSG).

**Methods:**

Female albino rats were randomly assigned across five groups: I (control), II (chemo-ablation), III (chemo-ablation + PMSG), IV (chemo-ablation + MSCs), and V (chemo-ablation + PMSG + MSCs). Postnatal oogenesis was assessed through measurement of *OCT4*, *OCT4A*, *Scp3*, *Mvh*, *Nobox*, *Dazl4*, *Nanog*, *Sca-1*, *FSHr*, *STRA8*, *Bax*, miR143, and miR376a transcript levels using qRT-PCR*.* Expression of selected key proteins were established as further confirmation of transcript expression changes. Histopathological examination and ovarian hormonal assessment were determined.

**Results:**

Group V displayed significant upregulation of all measured genes when compared with group II, III or IV. Protein expression confirmed the changes in transcript levels as group V displayed the highest average density in all targeted proteins. These results were confirmed histologically by the presence of cuboidal germinal epithelium, numerous primordial, unilaminar, and mature Graafian follicles in group V.

**Conclusion:**

VSELs can restore the postnatal oogenesis in chemo-ablated ovaries treated by BM-MSCs combined with PMSG.

## Introduction

Mammalian ovaries are responsible for competent mature oocyte formation. They are also responsible for the secretion of a variety of hormones, growth factors and cytokines that contribute to key signaling pathways of oogenesis and folliculogenesis. Premature ovarian failure (POF) therefore results in the loss of reproductive ability and early menopause [1]. POF is manifested by amenorrhea, sex steroid hormone deficiency with elevated (menopausal) levels of serum gonadotropins, before the age of 40 years [2]. It is not a rare condition with an occurrence of approximately 6% in women of less than 40 years of age [3]. Further, reflecting advanced treatment options, more than 80% of young cancer patients survive with infertility as a side effect [4]. Currently, there is no standard approach for preventing radiation/chemotherapy-induced ovarian failure. Cryopreservation is the only established and standard method for fertility preservation in young women with cancer [5]. Thus, it is essential to identify better strategies to prevent ovary dysfunction during chemotherapy and restore ovary function after chemotherapy.

Postnatal oogenesis has emerged as a key theme within female reproductive science. This has challenged established concepts within reproductive science including that all female mammals possess a constant pool of oocytes, which is not subjected to renewal during postnatal life [6]. However, experimental research in adult female mice verified that the imbalance between non-atretic follicle number and the rate of atresia failed to clarify the fixed number of germ cell theory [7]. This was accompanied by the display of mitotically active germ cells expressing meiotic markers within the adult ovary of mice. These, and additional, observations reveal the occurrence of potential postnatal oogenesis through the existence of germline stem cells in mice and higher animal species including human [8, 9].

The population of putative pluripotent stem cells (PSCs) within the ovarian epithelial surface of adults have been hypothesized to drive postnatal oogenesis [10, 11]. Neo-oogenesis requires germline stem cells (GSCs) in the ovarian surface epithelium (OSE) with the ability to differentiate into oocyte, granulosa phenotype, fibroblast-like cells, and in vitro, with the proper stimulation, into mesenchymal cell lineages. Endpoint indications ranging from oocyte quantification, genetic lineage tracing, and transplantation support a paradigm shift in reproductive biology that includes active renewal of oocyte-containing follicles throughout postnatal life [12]. Previous reports have indicated that a population of stem cells in the adult human ovaries may have the potential to develop into an oocyte-like, embryoid body-like structure via their differentiation into the three embryonic layers in vitro [13, 14]. Taken together, these observations offer a potential role for these PSCs in aiding infertile couples conceive. Nevertheless, there remains substantial concern around postnatal oogenesis and its challenge to the fixed germ cell pool theory [15].

Quiescent and active stem cell populations are evidenced across multiple organs including skin, gut, brain [16], and gonads. The gonadal stem cells consist of two distinctive populations, specifically, the relatively “quiescent” very small embryonic-like stem cells (VSELs), and the committed “active” progenitor stem cell that includes ovary germ stem cells (OGSCs) in the ovary [17] and spermatogonial stem cells in testis [18]. These two populations are distinguishable due to their size, expression of octamer-binding transforming factor 4 (OCT-4) isoforms, and their capacity to proliferate [19]. Ovarian stem cells have been identified in the ovaries of perimenopausal women (including a 60-year-old woman) with no naturally present follicles or oocytes [9] and aged mice [20]. However, ovarian microenvironment becomes unsuitable for ovarian stem cells to proliferate and differentiate. So, if we try to improve the microenvironment to become suitable for proliferation and differentiation of ovarian stem cells, the postnatal oogenesis will be improved [17].

Follicle-stimulating hormone (FSH) is a pleiotropic hormone manufactured by the pituitary that utilizes its action on mammalian ovaries through prompting proliferation, differentiation, and finally steroidogenesis within the granulosa cells of the developing pre-ovulatory follicles. However, the initial development of primordial follicles (PF) is believed to be FSH independent [21].

Mesenchymal stem cells (MSCs) are a well-characterized non-hematopoietic adult stem cell derived from a wide range of tissues including bone marrow. Though conventionally isolated from bone marrow, MSCs have also been found in various additional adult tissues including umbilical cord, adipose tissue, liver, placenta and dental pulp. MSCs have substantial promise in therapeutic regenerative medicine application with strong potential in non-autologous transplantation approaches, due to their lack of MHC class II expression [22]. In addition, MSCs have anti-inflammatory, antifibrotic, antiapoptotic, and immunomodulatory effects that are paracrine in nature and have been verified in various pre-clinical studies and clinical indications. These distinctive features have created suitability across numerous medical indications where therapeutic intervention, tissue engineering, and cell therapy sit as credible options [23]. Included amongst this list is their potential application in reproductive health disorders including premature ovarian failure as MSCs are described as partially ameliorating hormonal function, folliculogenesis, and architecture of chemotherapy damaged ovaries of albino rats [24]. Further assessment is required to explore the effectiveness of these cells in infertility treatment and reproductive science [25, 26].

Therefore, in the present study, we assessed the capability of VSELs to restore the postnatal oogenesis in chemo-ablated ovaries treated with BM-MSCs combined with gonadotropin. We observed that the gonadotropin alone positively affected VSEL proliferation but that successful differentiation to primordial cells required MSC-driven repair to the damaged microenvironment via granulosa cells.

## Materials and methods

### Experimental animals

Nulliparous and sexually mature female albino rats within the diestrus (DE) phase (180–200 g), aged 6 weeks, were purchased from the Experimental Animal Unit, Faculty of Veterinary Medicine, Benha University, Egypt. The different stages of the estrus cycle were examined via daily examination of vaginal smears where, concisely, the vaginal swabs were prepared by means of sterile PBS-dipped cotton swabs, fixed in 100% methanol, stained with Giemsa, and examined underneath the microscope (Nikon, Tokyo, Japan). Rats were bred and kept up in an air-conditioned animal house under specific pathogen-free conditions. All animals were accommodated in clean cages and given a standard diet and clean water ad libitum*.* Rats were exposed to the normal light/dark cycle (12-h light-dark cycle starting at 8:00 AM), room temperature (23 ± 3 °C) and free access to chow and water were allowed. This study was carried out in a strict accordance with the approvals in the Guide for the Care and Use of Laboratory Animals of the National Institutes of Health (NIH publication No. 85–23, revised 2011). All protocols were permitted by the institutional review board for animal experiments of the Faculty of Medicine, Benha University, Egypt (BUFM 3 January 2018).

### Preparation and tracking of BM-derived MSC

Rats BM-MSCs were prepared in the Stem Cell Unit, Central Lab, Faculty of Medicine, Benha University. BM-MSCs cells were flushed from the tibia and fibula of rat bones by phosphate-buffered saline (PBS) having 2 ml EDTA. The diluted sample was cautiously layered over Ficoll-Paque (Gibco-Invitrogen, Grand Island, NY), centrifuged for 35 min at 400 rpm and the higher layer aspirated leaving the mononuclear cell (MNC) layer at the interphase. This MNC layer was aspirated, washed twice in PBS having 2 ml EDTA and centrifuged for 10 min at 200 rpm at 10 °C. The pellet of cells was re-suspended in PBS/EDTA before being seeded into T25 flasks in minimal essential medium (MEM) supplemented with 15% fetal bovine serum (FBS) at 37 °C and 5% CO_2_. The adherent BM-MSCs were cultured with MEM supplemented with 30% FBS, 0.5%, streptomycin, penicillin and at 37 °C in 5% CO_2_ in air [27]. All cultures were tested via an inverted microscope; Leica DM IL LED with camera Leica DFC295 (Leica Microsystems CMS GmbH, Ernst-Leitz- Straße 17-37, Wetzlar, D-35578, Germany) [28].

### Immunophenotyping characterization of differentiated stem cells

BM-MSCs were primarily characterized by their adhesiveness, fusiform morphology, and through the recognition of the well known surface markers of rat BM-MSCs via flow cytometry. Subsequent the isolation, BM-MSC were passaged, viable cell counts recognized, and aliquoted individually at 1 × 10^6^ cells/mL per tube. BM-MSC were then incubated with 10 μL of directly conjugated monoclonal antibodies; CD34 PE (rabbit monoclonal; EP373Y, ab223930), CD90 PE (mouse monoclonal Antibody (HIS51), eBioscience, # 14-0900-81), and CD 105 PE (rabbit polyclonal antibody, CENTER E395; SAB1306487 Sigma-Aldrich) at 4 °C in the dark for 20 min; matched isotype controls were included for control purposes. Subsequent the incubation, 2 mL of PBS containing 2% FCS solution was added to each tube followed by centrifugation for 5 min at 2500 rpm, discarding of the supernatant, and resuspending in 500 μL PBS containing 2% FCS. Cell analysis was executed using CYTOMICS FC 500 Flow Cytometer (Beckman Coulter, Brea, CA, USA) and CXP software version 2.2 [29].

### In vitro adipogenic, chondrogenic, and osteogenic differentiation of MSCs

BM-MSCs were examined for their capability to undergo trilineage differentiation into adipocytes, osteoblasts, and chondrocytes. Passage 4 MSCs at a density of 5 × 10^3^ cells/cm^2^ were seeded into precoated coverglass situated within six-well plates and encouraged for 3 weeks with either adipogenic (HUXMA-90031; Cyagen Biosciences Inc., Guangzhou, China), osteogenic (#HUXMA-90021; Cyagen Biosciences Inc.), or chondrogenic (#HUXMA-90041; Cyagen Biosciences Inc.) differentiation media, respectively. The cells were then fixed in 10% formalin and stained with either Oil Red O, Alizarin red, or Alcian blue according to standard procedures [30].

### Labeling stem cells with green fluorescent protein (GFP)

BM-MSCs were transfected with non-integrating plasmids containing GFP (addgene, PET His6 GFP TEV LIC cloning vector (1GFP) (Plasmid #29663). One day earlier to the transfection, 5 × 10^5^ cultured cells were plated in 1 ml complete growth medium and BM-MSCs transfected with a single plasmid using the Nucleofector kit (Lonza, Verviers, Belgium) according to the manufacturer’s instructions [31]. BM-MSCs labeled with GFP were detected via a fluorescence microscope (Leica Microsystems CMS GmbH, Ernst-Leitz-Straße, Wetzlar, D-35578, Germany).

### Induction of ovarian ablation:

Rats were administered with 10 mg/kg busulfan daily for 4 days and 100 mg/kg cyclophosphamide on the first 2 days resulting in complete germ cell depletion. Busulfan (Sigma-Aldrich, Missouri) was dissolved in dimethyl sulfoxide (Sigma-Aldrich), diluted with an equal volume of water, and injected via the intraperitoneal route. Cyclophosphamide (Baxter, India) was dissolved in sterile injection-grade water and also injected via intraperitoneal route after 1 to 2 h of busulfan injection [32].

### Experimental design and treatment protocol

Fifty-six rats were divided randomly into five groups as follows:

- Group I (control group): Twenty-eight rats were subdivided equally into 4 subgroups (7 animals per group):
  - Subgroup a: Rats with no intervention.
  - Subgroup b: Rats were injected with dimethyl sulfoxide (Sigma-Aldrich), diluted with an equal volume of water, and injected via the intraperitoneal route.
  - Subgroup c: Rats were injected with sterile injection-grade water via the intraperitoneal route.
  - Subgroup d: Rats were injected intraperitoneally with sterile phosphate-buffered saline solution.
- Group II (chemo-ablation group; OA group): seven rats underwent induction of ovarian ablation by injecting busulfan 10 mg/kg daily for 4 days and 100 mg/kg cyclophosphamide in the first 2 days.
- Group III (OA + PMSG group): Seven rats were injected subcutaneously 5 IU PMSG (National Hormone & Peptide Program, Harbor-UCLA Medical Center, California) 1 month after treatment with busulfan and cyclophosphamide.
- Group IV (OA + BM-MSCs cell group): Seven rats underwent ovarian chemo-ablation, then after 1 month, the rats received a therapeutic delivery of MSC as a single intraperitoneal injection (1 × 10^6^ MCSs were suspended in 0.5 mL of phosphate-buffered saline).
- Group V (OA + PMSG+ BM-MSCs group): Seven rats underwent ovarian chemo-ablation, and then stem cells were injected by intraperitoneal route, as above, combined with a single subcutaneous injection of 5 IU (pregnant mare serum gonadotropin PMSG), after 1 month of chemo-ablation.

#### Sampling

Four weeks after gonadotropin administration and stem cell injection, all rats were anesthetized by sodium thiopental anesthesia (40 mg/kg; I.P.) after 12 h of fasting. The rats were fixed on an operating table and blood samples obtained from retro-orbital venous plexus using a fine-walled Pasteur pipette. This was followed by vascular perfusion fixation by using 10% buffered formol saline through the left ventricle. Following fixation, the ovaries of rats of all groups were dissected for both histopathological examination [hematoxylin and eosin (H&E) and immunohistochemically staining for proliferating cell nuclear antigen (PCNA) and FSHr] in addition to molecular analysis by real-time PCR and Western blot.

### Determination of serum FSH and estradiol E concentrations

Orbital blood was obtained under anesthetized conditions with chloral hydrate. The serum of rats was isolated via centrifugation at 4 °C and stored at − 80 °C until assay. The serum levels of FSH and estradiol E were assessed using ELISA kits (Uscn Life Science Inc., Houston, TX, USA) and were read on ELISA Reader (Thermo Fisher, Varioskan Flash, USA) following the manufacturers’ guidelines. Measurements were done in technical triplicate and the levels of FSH and estradiol E presented as nanograms per milliliter.

### Gene expression profile

Total RNA was extracted from the ovaries of all experimental groups using TRIzol (Invitrogen) according to the manufacturer’s instructions. The concentration and purity of extracted RNA were assessed by the Nano-Drop 2000C spectrophotometer (Thermo Scientific, USA). At absorbance ratio A260/A280, RNA purity for all samples was > 1.9. The integrity of RNA was confirmed on 2% agarose gel using a gel electrophoresis image (Gel Doc. Bio-Rad) according to [33]. Complementary DNA (cDNA) was synthesized for the target genes using SensiFast cDNA synthesis kits (Sigma Bioline, UK) according to the manufacturer’s instruction. NCode VILO miRNA cDNA Synthesis Kit (Invitrogen) was used for cDNA synthesis from miRNAs following the manufacturer’s recommendations using a T100 Thermal Cycler (Bio-Rad, USA).

Quantitative PCR was done using Maxima SYBR Green/ROX qPCR master mix (2x) (Thermo Scientific, USA) according to [34]. Primer pairs for selected target and reference genes (SCP3, synaptonemal complex protein 3; Mvh, mouse VASA homolog; Nobox, NOBOX oogenesis homeobox; DAZL, deleted In azoospermia like; Oct-4, octamer-binding transcription factor 4; Nanog, Nanog Homeobox; Sca-1, stem cell antigen-1; FSHr, follicle stimulating hormone receptor; Oct-4A, octamer-binding transcription factor 4 isoform A; Stra8, stimulated by retinoic acid 8; BAX; GAPDH, glyceraldehyde-3-phosphate dehydrogenase) were purchased from Genwez (New Jersey, USA) (Table 1). Each PCR reaction consisted of 500 ng per reaction of cDNA (except for NTC and cDNA control), 12.5 μl Maxima SYBR Green qPCR Master Mix (Maxima SYBR Green qPCR, Thermo Fisher Scientific), 0.3 μmol l^−1^ of each forward and reverse primer, 10 nmol l^−1^/100 Nm ROX Solution, nucleases-free water to a final volume of 25 μl. The reaction was completed in AriaMx real-time PCR (Agilent Technologies, USA) using a two-step protocol: initial denaturation at 95 °C for 10 min, then 40 cycles of denaturation at 95 °C for 15 s followed by annealing/extension at 60 °C for 60 s. A melting curve protocol was run at the end of the PCR by heating at 95 °C for 30 s followed by a 65 °C for 30 s and 95 °C for 30 s. The expression levels of target genes were normalized to the housekeeping gene glyceraldehyde-3-phosphate dehydrogenase (GAPDH) while the expression of miRNAs was normalized relative to U6 RNA as an internal housekeeping reference gene. Relative gene expression ratios (RQ) between treated and control groups were calculated using the formula: RQ = 2^−ΔΔCt^ [40]

**Table 1:** Primer sequences and accession number of the studied genes

Gene	Accession no.	Primers sequences (5′ → 3′)	Reference
*SCP3*	XM_032910940.1	F: TGTTGCAGCAGTGGGAACTGGAT	[35]
		R: CCATCTCTTGCTGCTGAGTTTCCA	
*Mvh*	XM_032898891.1	F: AGTGGAAGTGGTCGAGGTGGT	[26]
		R: TGCCGGTGGTGCATCATGTCC	
*Nobox*	NM_001192013.1	F: AGGGTGCTGAGAGGGTGGCAG	[36]
		R: GGCGATACTAGTGCCCCAGGAC	
*DAZL*	XM_032899416.1	F: GTGTGTCGAAGGGCTATGGAT	[35]
		R: ACAGGCAGCTGATATCCAGTG	
*Oct-4*	XM_032889059.1	F: CCTGGGCGTTCTCTTTGGAAAGGTG	[37]
		R: GCCTGCACCAGGGTCTCCGA	
*Nanog*	NM_028016.3	F: CAGGAGTTTGAGGGTAGCTC	[37]
		R: CGGTTCATCATGGTACAGTC	
*Sca-1*	NM_001310438.1	F: AGAGGAAGTTTTATCTGTGCAGCCC	[35]
		R: TCCACAATAACTGCTGCCTCCTGA	
*FSHr*	XM_021218040.1	F: TGGAGGCGGCAAACCTCTGAAC	[38]
		R: TCTGGCTTTGGCGAGCAGGTC	
*Oct-4A*	AH003838.2	F: CCATGTCCGCCCGCATACGA	[37]
		R: GGGCTTTCATGTCCTGGGACTCCT	
*GAPDH*	XM_032909104.1	F: ACCACAGTCCATGCCATCAC	[37]
		R: TCCACCACCCTGTTGCTGTA	
*Stra8*	XM_006236282.3	F: CAGCCTCAAAGTGGCAGGTA	Designed
		R: GGGATTTCCGTCTTGCAGGT	
*BAX*	XM_032913059.1	F: CGGCGAATTGGAGATGAACTGG	[39]
		R: CTAGCAAAGTAGAAGAGGGCAACC	

### Western blot

Anti-rabbit polyclonal antibodies against Oct-4 (Abcam, ab18976)), Oct-4A (C30A3) mAb #2840), Sca-1 (Abcam, ab95439), FSHr (Abcam, ab75200), and *β*-actin (Abcam, ab8226), and anti-mouse monoclonal against Nanog (Santa Cruz, Cat#sc8630), were used. Protein extraction was performed using ice-cold 1X cell lysis buffer containing 50 mmol Tris (SRL, Mumbai, India), 1 mmol EDTA (Fischer Scientific, New York), 150 mmol NaCl (Sigma-Aldrich), 1 mol sodium fluoride (Fischer Scientific, Qualigens, Mumbai), 0.1% sodium dodecyl sulfate (SDS; Fischer Scientific), 1% Triton X-100 (Sigma-Aldrich), 2 mmol phenylmethylsulfonyl fluoride (Sigma-Aldrich), and 4% protease inhibitor cocktail (Roche Diagnostics, Manheim, Germany). Whole ovaries were minced and homogenized by passing through 20G, 22G, and 26G hypodermic needles.

The subsequent cell lysates were agitated on ice for 30 min followed by centrifugation at 21,000*g* for 30 min to assemble the supernatant. Protein concentration was assessed by the Folin-Lowry method by means of a spectrophotometer (Beckman Coulter Inc, Indianapolis, Indiana). The extracted protein was incubated in Laemmli buffer for 10 min at 95 °C. Protein (50 mg loading) was resolved using 10% SDS (sodium dodecyl sulfate) polyacrylamide gel electrophoresis and transferred onto polyvinylidene difluoride membrane (Amersham Biosciences, Bucks, UK). The blot was blocked with 5% nonfat dry milk (NFDM) in 1% TBS with Tween 20 overnight at 4 °C. The membrane was incubated with anti-OCT-4, Oct-4A, Sca-1, Nanog, FSHr, and β-actin antibodies (1:500; Ab3209, Millipore) at room temperature for 2 h followed by incubation with goat anti-rabbit horseradish peroxidase-conjugated secondary antibody (1:5000, Millipore) for 2 h at room temperature.

Recognition was performed with Super Signal West Femto substrate (Thermo Scientific, Waltham, Massachusetts) on photographic films (Eastman Kodak Co, Rochester, New York). The later blot was stripped via stripping buffer (62.5 mmol Tris, 2% DS, 100 mmol *β*-mercaptoethanol) for 10 min at 60 °C to detect housekeeping protein. Actin was cast-off as housekeeping protein and detected using MAB1501 (Millipore) at a 1:5000 dilutions with human embryonic stem cell extract used as positive control. Afterward washing twice with 1X TBST, densitometric analysis of the immunoblots was done to calculate the amounts of OCT-4, Oct-4A, Sca-1, Nanog, FSHr, and *β*-actin against control sample by total protein normalization using Image analysis software on the Chemi Doc MP imaging system (Version 3) made by Bio-Rad (Hercules, CA).

### Histopathological analysis

Ovarian tissues were excised, fixed in 10% buffered formol saline, and managed as 4-6-μm-thick paraffin sections, followed by mounting on glass slides for H&E and positively charged slides for immunohistochemistry (IHC).

Half of the ovarian tissues were consumed for H&E staining. Next to the fixation sections were dehydrated with successive concentrations of ethanol and washed twice in distilled water followed by staining with hematoxylin and eosin (H&E). Lastly, the histological sections were examined and analyzed under a microscope (Leica DMR 3000; Leica Microsystem) by two blinded experienced investigators [41].

### Immunohistochemistry analysis

Paraffin sections were deparaffinized and hydrated. After blocking the endogenous activity of peroxidase using 10% hydrogen peroxide, the sections were incubated with primary rabbit polyclonal antibodies against PCNA (proliferating cell nuclear antigen) (SAB4502103; 1:400 dilutions; Sigma-Aldrich, St Louis, Missouri, USA) and rabbit polyclonal antibodies against FSHr (F3929; 1:100 dilutions; Sigma-Aldrich, St Louis, Missouri, USA). Rabbit polyclonal antibodies against OCT4 (# PA5-27438; 1:100-1:1000, Thermo Fisher Scientific). Also, we used polyclonal rabbit antibodies against CD105 (SAB1306487; 1:10-1:50, Sigma-Aldrich) to investigate homing of MSCs in ovarian tissues. Then, after washing with phosphate buffer, the secondary antibody was applied (biotinylated goat anti-rabbit). The slides were incubated with labeled avidin–biotin peroxidase, which binds to the biotin on the secondary antibody. The site of antibody binding was visualized after adding (diaminobenzedine) chromogen, which is converted into a brown precipitate by peroxidase [42].

### Morphometric study

The mean area % of PCNA, FSHr, nuclear OCT4, and CD105 immuno-expression were quantified for five images from five non-overlapping fields from each rat of each group using the Image-Pro Plus program version 6.0 (Media Cybernetics Inc., Bethesda, Maryland, USA).

### Statistical analysis

Statistical analysis was performed using the statistical software package SPSS for Windows (Version 16.0; SPSS Inc., Chicago, IL, USA). Differences between groups were evaluated using one-way analysis of variance (ANOVA; F) and Kruskal Wallis test (χ^2^) to compare more than two groups regarding parametric and non-parametric data, respectively, followed by post hoc analysis to detect differences in pairs. For each test, all data are expressed as the mean ± standard deviation (SEM), and a *P* value < 0.05 was considered significant.

## Results

### Confirmation of BM-MSC isolation

**Fig. 1:**
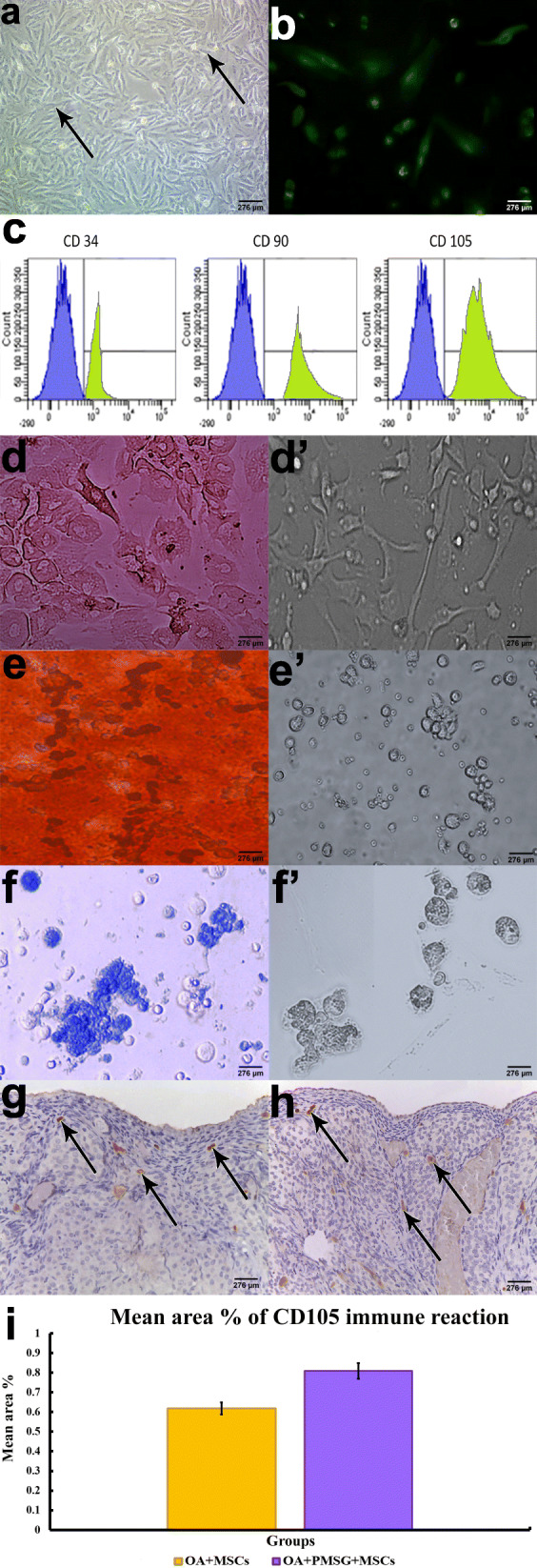
**a** An inverted microscope micrograph from primary culture of mesenchymal stem cells (black arrow). **b** Fluorescent microscopic image demonstrating fluorescence of MSCs labeled with GFP 2 weeks after implantation. **c** Flow cytometry analysis of surface antigens of MSCs (blue is the isotype, green is the detected antigen); CD34 0.7%, CD90 92.5%, and CD105 98.7%. **d** Osteogenesis differentiation stained with Alizarin red stain and its control. **e** Adipogenesis differentiation stained with Oil Red O stain and its control. **f** Chondrogenesis differentiation stained with Alcian blue stain and its control. **g** CD105 immuno-expression in group VI. **h** CD105 immuno-expression in group V. **i** Histogram representing the mean area percentage of CD105 immunoreaction in treated groups (group IV and V). The significant differences against chemo-ablated group indicated by a “*” for *p* < 0.05 and a “**” for *p* < 0.01. Data are shown as mean ± S.E.M, *n* = 7

BM-MSCs were recognized primarily after 2 weeks’ isolation in culture via an inverted microscope as spindle-shaped cells among rounded cells (Fig. 1a) and intraperitoneal injected BM-MSCs labeled with GFP were observed using a fluorescent microscope (Fig. 1b). Cell surface marker expression confirmed BM-MSC identity via CD90 (92.5% positive expression), CD105 (98.7% positive), and CD34 (0.7% positive) being consistent with the ISCT MSC guidelines [43] (Fig. 1c). Confirmation of the adipogenic, osteogenic, and chondrogenic differentiation was also established (Fig. 1d–f). The localization and homing of BM-MSCs were assessed in MSC-treated groups (group IV and V) by assessing the immune-expression of MSC surface marker (CD105) in ovarian tissues (Fig. 1g–i).

### Gonadotrophin and BM-MSC supplementation promote elevated serum estradiol E levels

**Fig. 2:**
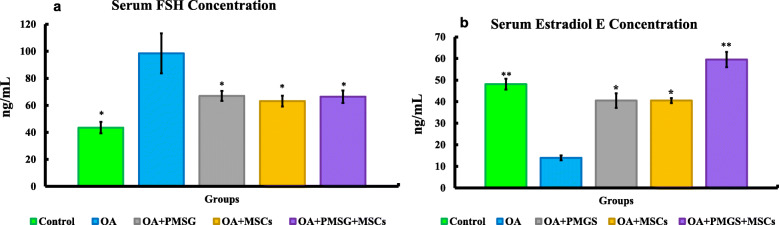
Serum FSH (**a**) and estradiol E (**b**) concentrations after rat’s scarification. The significant differences against chemo-ablated group indicated by a “*” for *p* < 0.05 and a “**” for *p* < 0.01. Data are shown as mean ± S.E.M, *n* = 7

Following on from ovarian chemo-ablation by combined administration of busulfan daily for 4 days and 100 mg/kg cyclophosphamide on the first 2 days, we sought to determine whether gonadotrophin and BM-MSCs carried with them a therapeutic effect. Following on from chemo-ablation, and 1 month after therapeutic intervention by gonadotrophin and MSCs, we measured serum FSH and estradiol E concentrations using enzyme-linked immunosorbent assay (ELISA) (Fig. 2). Serum FSH concentration was significantly (*P* < 0.05) higher in group II (OA group) when compared with all other experimental groups (Fig. 2a). No other groups displayed significant elevation of serum FSH above control levels.

Estradiol E, a major female sex hormone, is produced by the growing ovarian follicles. Its level was significantly (*P* < 0.05) decreased in the serum of group II (OA group) when compared with all other groups. Estradiol E levels in group V (OA + PMSG+ BM-MSCs group), treated with both gonadotrophin and MSCs, were significantly higher (*P* < 0.05) than all other groups (Fig. 2b).

### A distinct molecular signature associates with gonadotrophin and BM-MSC-supplemented ovaries following on from chemo-ablation

**Fig. 3:**
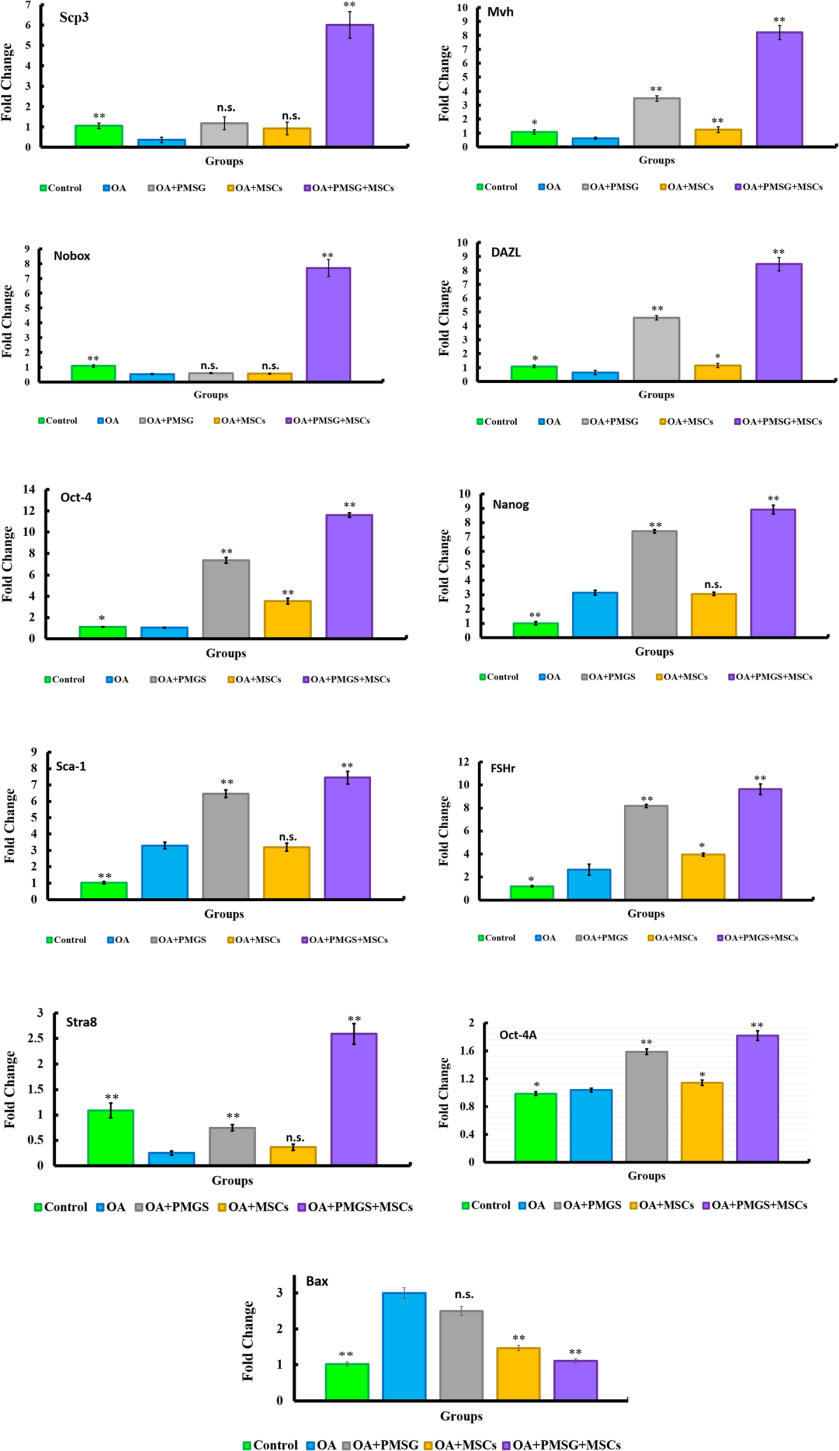
Quantitative analysis for relative expression of Scp3, Mvh, Nobox, Dazl, Oct-4, Nanog, Sca-1, FSHr, Stra8, Oct-4-A, and BAX. The significant differences against chemo-ablated group indicated by a “*” for *p* < 0.05 and a “**” for *p* < 0.01. Data are shown as mean ± S.E.M, *n* = 7

We next explored expression of a range of transcripts recognized as ovarian mediators (Fig. 3). Nanog, Sca-1, and Oct-4a were upregulated in group II (OA group) compared to control group (*P* < 0.05), while Scp3, Mvh, Nobox, DAZL, Stra8, and Oct-4 were all downregulated in group II (OA group) compared to control group (*P* < 0.05). Group III (OA + PMSG group) showed upregulation of Mvh, DAZL, Oct-4, Nanog, Sca-1, FSHr, Oct-4a, and Stra8 when compared with group II (OA group) (*P* < 0.05). In contrast, Scp3 and Nobox were unchanged between group III (OA + PMSG group) and group II (OA group). Group IV (OA + BM-MSCs group) showed upregulation in Mvh, DAZL, Oct-4, and FSHr expression (*P* < 0.05) and no change in Scp3, Nobox, Stra 8, Nanog, Sca-1, and Oct-4a when compared with group II (OA group). Group V (OA + PMSG+ BM-MSCs group) displayed significant upregulation of all measured genes when compared with group II (OA group) and to a greater extent than seen in other groups (*P* < 0.05). In contrast to the general trend noted above BAX was upregulated in group II (OA group) when compared to the control group (*P* < 0.05). Further, both groups IV (OA + BM-MSCs group) and V (OA + PMSG+ BM-MSCs group) displayed downregulation of BAX expression when compared to either group II or group III (*P* < 0.05).

### miR-143 and miR-376a have distinct roles in primordial follicle pool formation and maintenance

**Fig. 4:**
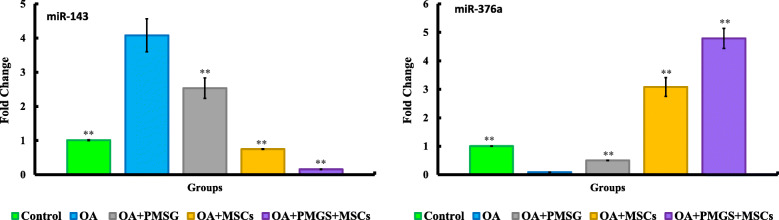
Quantitative analysis for relative expression of miR-143 and miR-376a. The significant differences against chemo-ablated group indicated by a “*” for *p* < 0.05 and a “**” for *p* < 0.01. Data are shown as mean ± S.E.M, *n* = 7

miR-143 is described as playing a role in inhibition of primordial follicle formation by suppressing pregranulosa cell proliferation and downregulation of cell cycle-related genes [44] Significant upregulation of miR-143 expression was noted for group II (OA group) vs. group I (control group). Consistent with above, both groups IV (OA + BM-MSCs group) and V (OA + PMSG+ BM-MSCs group) showed downregulation of miR-143 when compared with group II, and group I for group V (OA + PMSG+ BM-MSCs group) (*P* < 0.05) (Fig. 4).

miR-376a is described as a stimulator of ovarian surface epithelial cell proliferation. Expression of ovarian miR-376a was downregulated in group II (OA group) when compared with the control group (*P* < 0.05) while the control group and group III (OA + PMSG+ group) displayed comparable expression levels. Groups IV (OA + BM-MSCs group) and group V (OA + PMSG+ BM-MSCs group) both displayed a significant upregulation of miR-376a when compared with either groups I or II (*P* < 0.05) (Fig. 4).

### Western blotting of selected key proteins as a confirmation of transcript expression changes

**Fig. 5:**
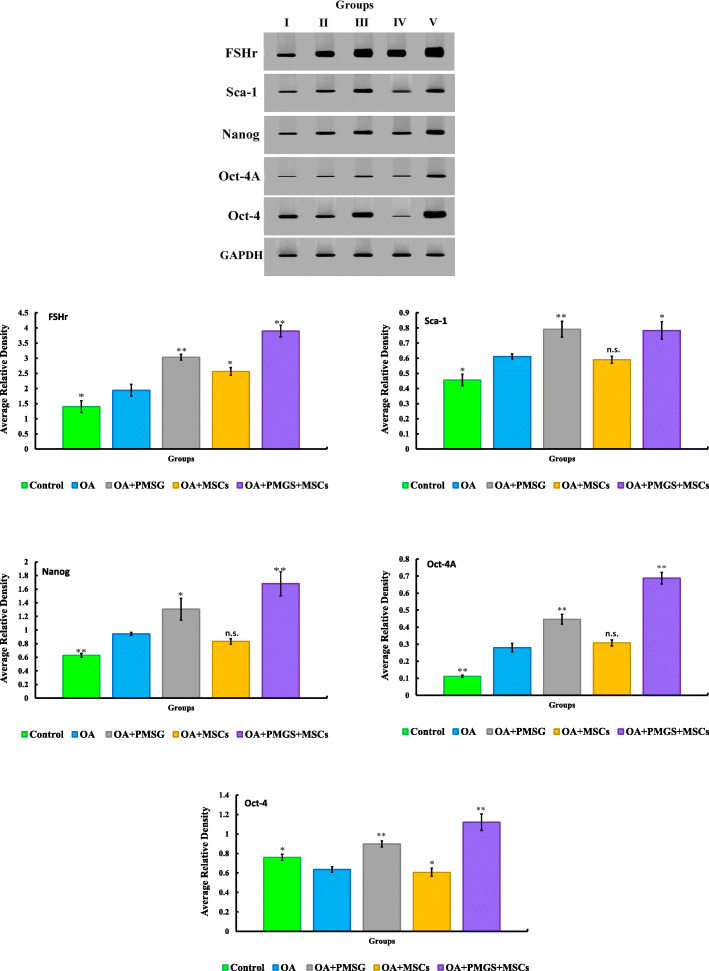
Western blot for Oct-4, Oct-4A, Sca-1, Nanog, FSHr, and GAPDH. Quantified using image analysis software on the Chemi Doc MP imaging system

Western blotting and quantitative analysis confirmed that Nanog, Sca-1, Oct-4a, and FSHr proteins were upregulated in group II (OA group) vs. the control group (*P* < 0.05), while Oct-4 protein was downregulated in group II vs. group I (*P* < 0.05). Group IV (OA + BM-MSCs group) showed upregulation of Oct-4 and FSHr protein (*P* < 0.05) and no change in Nanog, Sca-1, and Oct-4a when compared with group II (OA group). Group II (OA + PMSG group) and group V (OA + PMSG+ BM-MSCs group) showed higher expression of all five proteins when compared with group II (Fig. 5).

### Histological findings underlying postnatal oogenesis of chemo-ablated ovaries treated with gonadotrophin and BM-MSCs

No histological differences were observed upon examination of ovarian sections from the control subgroups. Therefore, the results of subgroup Ia were used to represent the control group.

#### H&E results

**Fig. 6:**
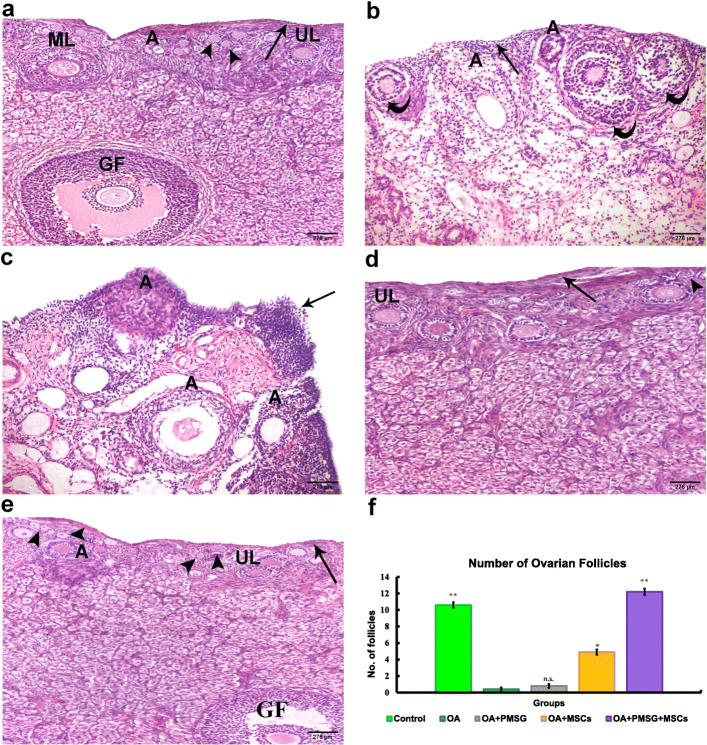
Representative photomicrographs of hematoxylin and eosin-stained sections of the different experimental groups. **a** Group I (control group) showed the germinal epithelium (thin arrow), primordial follicles (arrowheads), unilaminar (UL), and multilaminar (ML) primary follicles. Several atretic follicles (A) and a mature Graafian follicle (GF) were also observed. **b** Group II (OA group) showed flat ovarian surface (germinal) epithelial cells (thin arrow). No primordial follicles were observed. Secondary follicles (curved arrows) showed shrunken oocytes and wide separation of the surrounding granulosa cells. **c** Group III (OA + PMSG group) showed an area of prominent germinal epithelium stratification. No primordial follicles were seen, and the follicles appear atretic (A). **d** Group IV (OA + BM-MSCs group) showed flat germinal epithelium (thin arrow), numerous primordial (arrowheads), and unilaminar (UL) follicles. **e** Group V (OA + PMSG+ BM-MSCs group) showed cuboidal germinal epithelium (thin arrow), numerous primordial (arrow heads), unilaminar (UL), and a mature Graafian follicle (GF). **f** Histogram representing the mean number of ovarian follicle in all experimental groups. The significant differences against chemo-ablated group indicated by a “*” for *p* < 0.05 and a “**” for *p* < 0.01. Data are shown as mean ± S.E.M, *n* = 7

Group I (control group) revealed a normal ovarian structure. The surface of the ovaries was covered by germinal epithelium consisting of a single layer of cuboidal cells. Follicles in different stages of development were observed in the cortex. Primordial follicles were found beneath the germinal epithelium and consisted of primary oocytes surrounded by a single layer of squamous epithelium. The oocytes of the unilaminar primary follicles were surrounded by a single layer of cuboidal granulosa cells while those of the multilaminar primary follicles were surrounded by multiple layers of granulosa cells. Several atretic follicles were seen in addition to mature Graafian follicles (Fig. 6a). Ovaries from group II (OA group) demonstrated prominent histological alterations in the form of flat germinal epithelium, a marked reduction in primordial follicles, and severe atresia of the existing follicles. Secondary follicles had shrunken oocytes and wide separation of their surrounding granulosa cells (Fig. 6b). Treatment with gonadotropin alone in group III (OA + PMSG group) induced areas of prominent stratification of the germinal epithelium without primordial follicles, and follicles that appeared atretic (Fig. 6c). Group IV (OA + BM-MSCs group) ovaries displayed flat germinal epithelium with primordial and unilaminar follicles in the cortex (Fig. 6d). In group V (OA + PMSG+ BM-MSCs group), recipient of combined gonadotropin and MSC treatment, cuboidal germinal epithelium, numerous primordial, unilaminar, and mature Graafian follicles were observed (Fig. 6e).

#### Immunohistochemistry results

##### PCNA immunohistochemistry

**Fig. 7:**
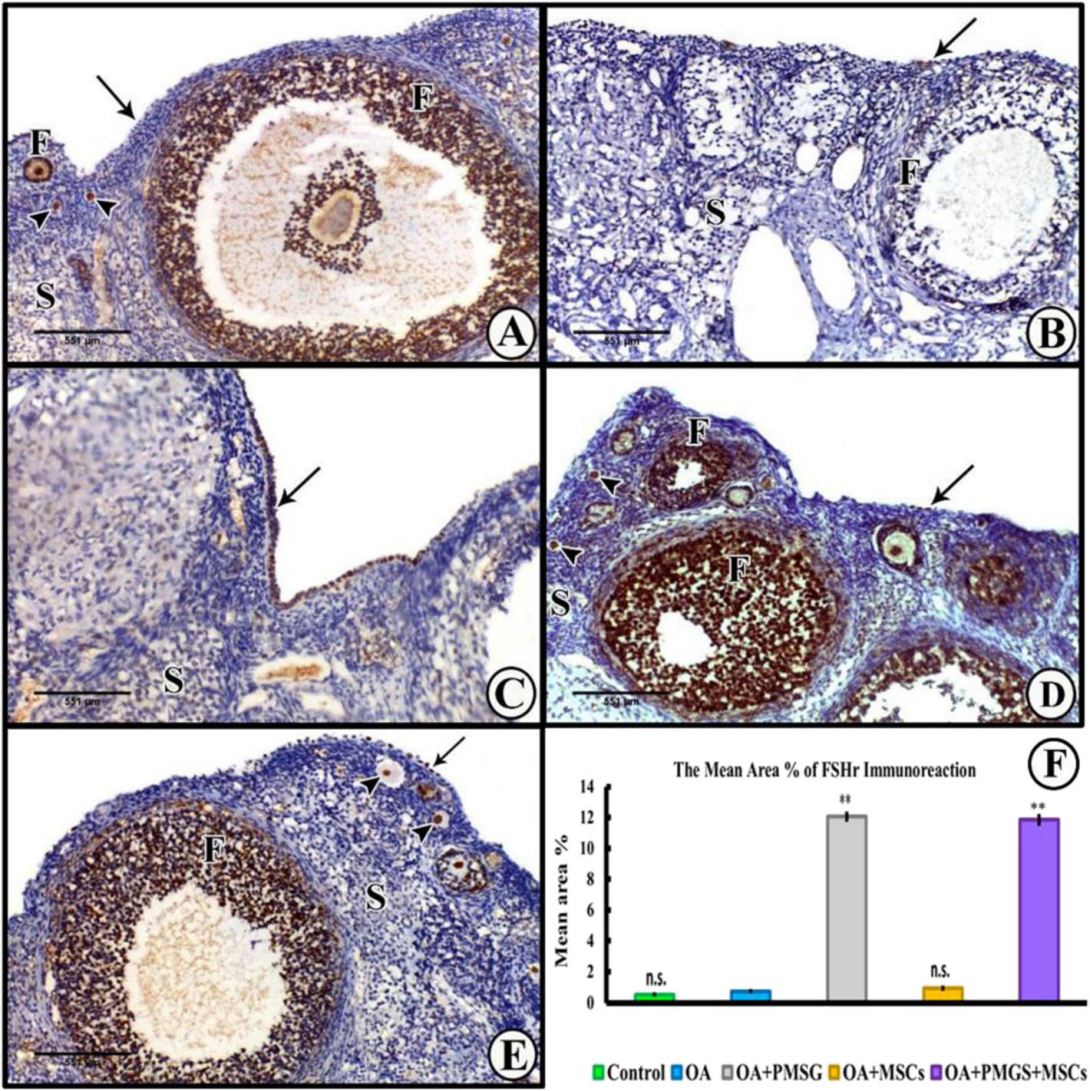
Representative photomicrographs of FSHr immunoreaction sections of the different experimental groups. **a** Group I (control group) showed a very mild positive nuclear reaction in a few of the surface epithelial cells. **b** Group II (OA group) showed limited FSHr nuclear expression in select surface epithelial cells. **c**, **d** Group III and group IV (OA + PMSG group and OA + BM-MSCs group) revealed a moderate FSHr expression in stratified surface epithelial cells. **e** Group V (OA + PMSG+ BM-MSCs group) resulted in a strong FSHr expression in the majority of surface epithelial cells. **f** Histogram representing the mean area percentage of FSHr immunoreaction in all experimental groups. The significant differences against the chemo-ablated group indicated by a “*” for *p* < 0.05 and a “**” for *p* < 0.01. Data are shown as mean ± S.E.M, *n* = 7

Group I (control group) revealed no evidence of nuclear PCNA in either the surface epithelial or stromal cells while positive labelling was observed in the follicular cells and the oocytes of the primordial follicles (Fig. 7a). Group II (OA group) displayed an absence of labelling in the surface epithelial, follicular, and stromal cells (Fig. 7b). Group III (OA + PMSG group) displayed strong immunolabelling in the stratified surface epithelial cells but none in either the follicular or stromal cells (Fig. 7c). Group IV (OA + BM-MSCs group) revealed an element of positive labelling in the surface epithelial and stromal cells while a positive reaction was observed in the follicular cells (Fig. 7d). Finally, group V (OA + PMSG+ BM-MSCs group) displayed positive immunolabelling in the surface epithelial and follicular cells as well as the primary oocytes of the primordial follicles but not in the stromal cells (Fig. 7e). The histogram representing the area percentage of PCNA in all groups (Fig. 9f).

##### FSHr immunohistochemistry

**Fig. 8:**
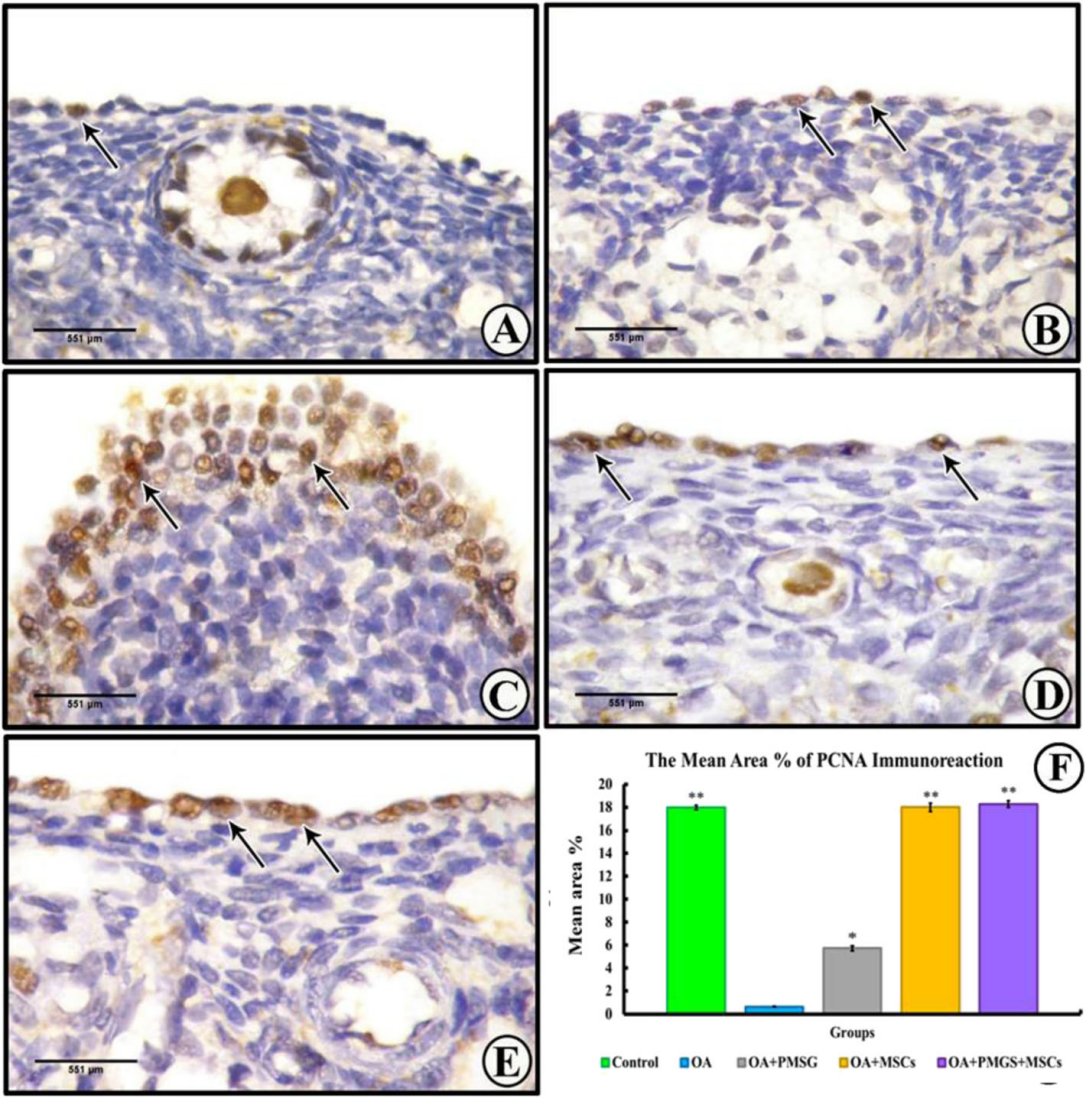
Representative photomicrographs of PCNA immune-stained sections of the different experimental groups. **a** Group I (control group) showed a negative nuclear reaction in the surface epithelial cells (thin arrow) and stromal (S) cells and a positive reaction in the primary oocytes of primordial follicles (arrowheads) and the follicular cells (F). **b** Group II (OA group) showed negative reaction in the surface epithelial cells (thin arrow), follicular (F), and stromal (S) cells. **c** Group III (OA + PMSG group) showing an intense reaction in the stratified surface epithelial cells (thin arrows) with a negative reaction in the follicular (F) and stromal (S) cells. **d** Group IV (OA + BM-MSCs group) showed a negative reaction in the surface epithelial cells (thin arrow) and stromal(S) cells and a positive reaction in the follicular (F) cells. **e** Group V (OA + PMSG+ BM-MSCs group) showed a positive reaction in the surface epithelial cells (thin arrow), primary oocytes of the primordial follicles (arrow heads), and follicular (F) cells with a negative reaction in the stromal (S) cells. **f** Histogram representing the mean area percentage of PCNA immunoreaction in all experimental groups. The significant differences against chemo-ablated group indicated by a “*” for *p* < 0.05 and a “**” for *p* < 0.01. Data are shown as mean ± S.E.M, *n* = 7

**Fig. 9:**
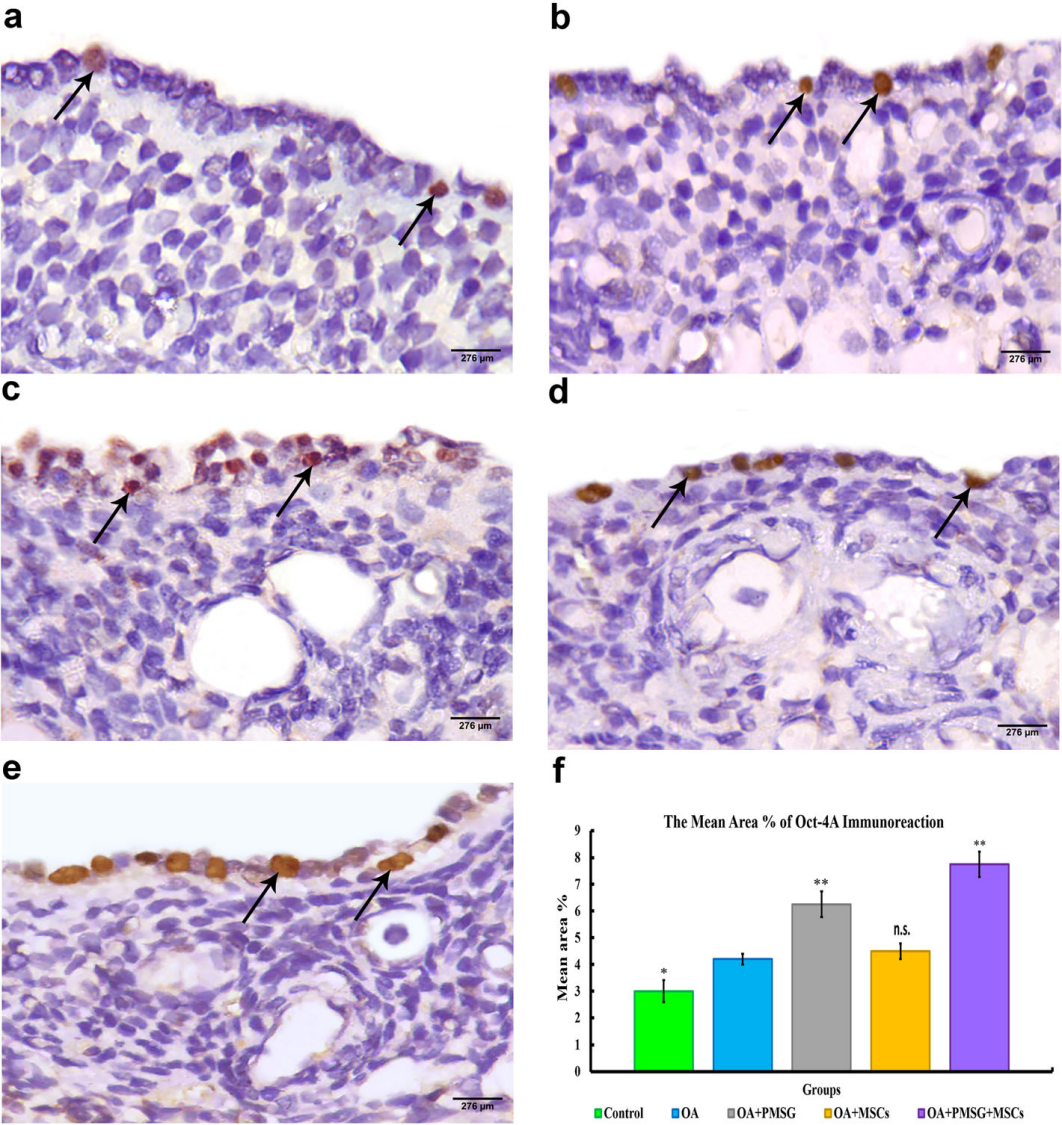
Representative photomicrograph of OCT4-A immunoreaction sections of different experimental groups. **a** Group I (control group) displayed minimal nuclear immuno-expression of OCT4-A in the surface epithelial cells. **b** Group II (chemo-ablated, OA group) showed mild nuclear OCT4-A immuno-expression in the surface epithelial cells. **c** Group III (OA + PMSG group) showed moderate nuclear OCT4A immuno-expression in the surface epithelial cells. **d** Group IV (OA + BM-MSCs group) showed minimal OCT4-A immuno-expression. **e** Group V (OA + PMSG+ BM-MSCs group) showed extensive immuno-expression of nuclear OCT4-A in the surface epithelial cells. **f** Histogram representing the mean area percentage of OCT4-A immunoreaction in all experimental groups. The significant differences against the chemo-ablated group indicated by a “*” for *p* < 0.05 and a “**” for *p* < 0.01. Data are shown as mean ± S.E.M, *n* = 7

Groups I (control group) and group II (OA group) displayed low levels of FSHr-labelling in the surface epithelial cells (Fig. 8a,b). Groups III (OA + PMSG group) and group IV (OA + BM-MSCs group) revealed a moderate reaction in stratified surface epithelial cells (Fig. 8c,d). While in the combined treatment recipient group V (OA + PMSG+ BM-MSCs group), a strong labelling was detected in many of the surface epithelial cells (Fig. 8e). The histogram representing the area percentage of FSHr in all groups (Fig. 9f).

##### OCT4-A immunohistochemistry

Groups I (control group) displayed minimal nuclear immuno-expression of OCT4-A in the surface epithelial cells indicating low level of VSELs in ovarian surface epithelial cells (Fig. 9a). While in group II (OA group), the VSELs resist chemotherapy giving mild nuclear immune-expression of OCT4-A in ovarian surface epithelial cells (Fig. 9b). In group III (OA + PMSG+ group), there were moderate nuclear OCT4-A immuno-expression compared to control group indicating increased percentage of VSELs in ovarian surface epithelial cells **(**Fig. 9c). While in BM-MSC-treated group (group IV), the nuclear OCT4-A immuno-expression was minimal indicating no change in VSEL percentage within ovarian surface epithelial cells compared to the chemo-ablated group **(**Fig. 9d**).** The nuclear OCT4-A immuno-expression in group V (combined therapy of BM-MSCs and gonadotropin) were severely increased compared to chemo-ablated group indicating increased number of VSELs in ovarian surface epithelial cells **(**Fig. 9e**).** The histogram representing the area percentage of OCT4-A in all group **(**Fig. 9f**).**

## Discussion

Until recently, fertility preservation protocols in women have been constrained by the theory that the oocyte reserve, endowed at birth, was fixed and indispensable. This theory was challenged by a report detailing the identification of mitotically active germ cells in postnatal mice ovaries capable of supporting de novo oogenesis and folliculogenesis throughout adult life [7, 45]. A large body of evidence has now verified postnatal oogenesis in mammals [46, 47], and the characteristic features and functional properties of the germ cells responsible for continued oocyte formation [48]. These cells, termed female germline or oogonial stem cells, have been detected and separated for studying, adult ovarian tissue of mice [47, 49], rats [50], cows [51], pigs [52], nonhuman primates [53], and women [18, 49, 54].

Cyclophosphamide and busulfan were used to induce ovarian chemo-ablation in rats. As ovaries express the enzymes necessary estrogen for the synthesis of [55] and that the majority of E2 is formed within the granulosa cells during the development of follicles under the effect of FSH and LH [56], these were utilized to establish chemo-ablation and recovery. Ovarian chemo-ablation was confirmed in group II by decreased serum estradiol E2, increased FSH, and histopathological examination that indicated decreased numbers of primordial follicles and severe atresia of the existing follicles, and flat germinal epithelium with little or no PCNA presence across surface epithelia, follicular, or stromal cells. Further, epithelial surface cells displayed FSHr expression indicating the potential presence of limited amounts of VSELS [32] These observations are consistent with reports detailing the depletion of primordial follicles and follicular atresia with extreme diminishing in ovarian function and eventual failure, after chemotherapy in rats [57]. The mechanism underpinning the primordial follicles loss in response to chemotherapy is not well understood. It might be attributed to an accelerated process of natural ovarian aging due to a direct cytotoxic influence on oocytes and a portion of primordial follicles, impeding folliculogenesis [58]. An alternative explanation to primordial follicle depletion after chemotherapy might be attributed to damaged cortical blood vessels and neovascularization. As the blood supply to the ovary is an end-artery system, therefore, congestion of blood vessels and marked thickening of the wall of blood vessels with obliteration of their lumina will result in local ischemia, destroying regions of the normal ovarian cortex with loss of primordial follicles [7].

In agreement with previous observations, we report the existence of two populations of potential stem cells in the adult mouse ovary [23, 59]. They are VSELs demonstrated by expression of nuclear OCT-4, SCA, and Nanog, and progenitors termed OGSCs characterized by cytoplasmic OCT-4. Control ovaries had reduced Oct-4A expression compared to total Oct-4, implying less VSEL presence and not OGSCs. On the other hand, chemo-ablated ovaries displayed less Oct-4 than Oct-4A transcript while the Oct-4A to Oct-4 ratio was higher than in controls, implying a predominance of VSELs. This is consistent with previous studies which revealed that chemotherapy destroyed, as expected, the ovarian follicular reserve and the potential OGSCs [23, 60]. VSELs may have survived due to their quiescence and silenced mitogenic growth response signaling pathways [32].

MicroRNAs (miRNAs) are linked to steroidogenesis, ovarian gonadal development, apoptosis, and mammalian ovulation [61–63]. Specific miRNAs in follicular development, mir-143, and miR-376a, are major negative and positive regulators of the formation and maintenance of the primordial follicle pool [64]. miR-143 is reported to inhibit the formation of mouse primordial follicles by downregulation of expression of cell cycle genes and suppressing the proliferation of pregranulosa cells [44]. This is potentially attributable to its ability to mediate the proliferative signaling pathway of FSH by FSHR targeting and the resultant inhibition of estradiol production and granulosa cell proliferation. Decreased expression of miR-143 in the OA + PMSC group is in broad agreement with earlier reports detailing the inhibition of miR-143 by FSH and TGF-β [44]. miR-376a stimulates ovarian proliferation as a positive regulator for formation and maintenance of the primordial follicle pool [64]. The expression of ovarian miR-376a was downregulated in the OA group when compared with the control group. The overexpression of ovarian miR-376a in the OA + PMSG + MSCs group mirrors high proliferation rates of primordial follicles as well as the reduction of oocyte apoptosis. Concomitant with these results, Zhang et al. [44] reported that the miR-376a controls primordial follicle assembly via controlling PCNA expression, a gene formerly reported to control primordial follicle assembly via controlling oocyte apoptosis in the ovarian mouse.

We demonstrated increased serum FSH and estradiol in group III (OA + PMSG group) in comparison to chemo-ablated group, as the VSELs were stimulated by PMSG treatment (indicated by increased Oct-4A, Nanog, and FSHr) expression, underwent proliferation (indicated by increased PCNA staining and Oct-4A expression), and further differentiation into germ cells (indicated by increased expression of total Oct-4, MVH, and DAZl) resulting in initiation of meiosis (indicated by increased expression of Stra-8, which is specifically expressed gene in mammalian germ cells. It is necessary for the initiation of meiosis and is expressed by ovarian germ cells just before the initiation of meiosis [10]. However, germ cells did not enter meiosis, with SCP3 neither expressed nor identified at the protein level in any of the experimentations. These results are in agreement with Bhartiya et al. [65] who recently reviewed the possible role of stem cells, FSH, and ovarian biology [65]. FSH increased proliferation of mouse OSE through oncogenic pathways activation in surface epithelial cells [65]. These oncogenic pathways include AKT, Birc5, Cdk2, Cdk4, and Cdkn2a [66]. Similarly, ovarian stem cells of sheep express FSH receptors and experience clonal expansion and proliferation in response to FSH through the novel FSHr transcript R3 [67]. Likewise, Sriraman et al. [68] reported that FSH treatment induced mouse ovarian stem cell proliferation and clusters formation and further differentiation into germ cells. These findings were confirmed histologically by prominent stratification of the germinal epithelium; however, no primordial follicles were observed, and the follicles remained atretic in appearance. Further, increased FSHr expression in group III (OA + PMSG group) compared to group II with accompanied PCNA expression was observed in the stratified surface epithelial cells and not the stromal and follicular cells. Nevertheless, the germ cells in chemo-ablated ovaries faced meiotic block, which is not overcome by FSH alone, and hence they proposed the requirement for additional factors. It is expected that in women with primary ovarian insufficiency and high levels of FSH, stem cells may experience proliferation and initial differentiation. Nevertheless, primordial follicle assembly and haploid oocyte formation may necessitate additional factors, that are not available in the compromised somatic niche (as a result of chemotherapy).

The apparent inability of VSELs to undergo differentiation may be a consequence of the compromised somatic niche as a result of treatment [4]. To attempt to overcome this, we provided an additional supplemental transplantation of MSCs with the aim of aiding niche restoration to support further differentiation of VSELs into gametes. Several studies of transplanted stem cells into a particular microenvironment have shown their stimulation by the niche. Micro-environmental stimuli, in turn, can trigger the paracrine secretion of growth factors which energize the regeneration of the surrounding tissue and potentially drive differentiation of both themselves and resident tissue-specific cell [69].

We have demonstrated higher FSHr mRNA and protein expression in group IV versus group II, suggesting that MSC administration promoted an increase in the cells number, as these, alongside VSELs, express FSHr [68]. It remains unclear if the MSCs differentiated into granulosa cells or the secreted trophic factors from MSCs enabled a revitalization of the damaged endogenous granulosa cells. However, no evidence was apparent to suggest that MSCs differentiated into oocytes in recipient rats as evidenced by the lack of the premeiotic (Stra8) and the meiotic (SCP3) markers, in agreement with the demonstration that MSCs did not create de novo germ cells (eggs) within implanted (donor) tissues [46]. Notwithstanding, the number of primordial and growing follicles after MSC treatment was not equivalent to the greater numbers present in the age-matched control group. Histological observation confirmed a flat germinal epithelium with primordial and unilaminar follicles in the cortex. FSHr expression was moderate while PCNA expression was detected in only a few surface epithelial and stromal cells with greater levels observed in the follicular cells. These observations demonstrated that MSC administration did not completely restore ovarian function to normal levels instead having a role limited to rescue of follicles experiencing early atresia or alternatively protecting quiescent primordial follicles from the opposing effects of chemotherapy in microenvironment of ovaries [70].

Folliculogenesis necessitates a cautiously orchestrated crosstalk between germ cells and surrounding somatic cells [71]. We found that combined injection of MSCs and PMSG caused a significant increase in the number of ovarian follicles and corpora lutea that was accompanied by restoration of E2 and FSH expression. This is in broad agreement with previous studies which found that the MSC-treated group upregulated proteins involved in epigenetic regulation, transcription, protein modification, and cell signaling [10, 72]. MSCs repaired the damage of ovarian tissues through the growth of granulosa cells that are stimulated by these pathways.

In addition to promoting repair of damaged ovarian cells, a combined injection of MSCs and PMSG dramatically reduced apoptosis of granulosa cells in the developing follicle. This reduction in apoptotic rate was accompanied by decreased BAX expression when compared to the chemo-ablated group.

Previous reports have suggested that MSCs inhibit apoptosis by the secretion of stanniocalcin-1 and other paracrine factors [73]. Therefore, we hypothesize that the combined theory applied above has reactivated host oogenesis, which is otherwise impaired following chemotherapy. The supplementation of MSCs and PMSG following on from chemo-ablation resulted in increased mRNA of germ cell-specific markers (OCT4, Dazl, MVH) suggesting increased differentiation of VSELs to germ cells. Moreover, meiosis was sustained by the expression of premeiotic (Stra8) besides meiotic (Scp3) markers. Furthermore, increased expression of the oocyte-specific marker (Nobox), which is expressed plentifully in primordial plus primary follicular oocytes [74], and the antiapoptotic factor in granulosa cells and folliculogenesis marker (FSHr) were noted [4]. These data suggest that combined injection of MSCs and PMSG amplifies the ovarian stem cell differentiation process as well as meiosis creating new oocytes, which are subsequently collect by granulosa cells to collect as primordial follicles, supporting the earlier proposal of postnatal oogenesis [75]. Noticeably, primordial follicles at birth are normally arrested in the diplotene stage of prophase 1 of meiosis while Scp3 expression constrained to former stages, i.e., zygotene and pachytene stages [7, 36]. Therefore, expression of Stra8 and Scp3 in the adult ovaries reflected evidence in supporting postnatal oogenesis.

Proliferating cell nuclear antigen (PCNA), an auxiliary protein of DNA polymerase enzymes, is used as a standard marker in proliferative cells [76]. Here, after chemotherapy, the vast majority of surface epithelial cells lacked PCNA expression. Following on from MSC and PMSG therapy, expression of PCNA was restored to surface epithelial cells when compared to the chemo-ablated group and the group treated with PMSG alone. Thus, MSCs possibly migrated from the bone marrow toward the ovary, to become a source of germline stem cells capable of regenerating the population of primordial follicles. Besides, they improve the microenvironment needed for oogenesis. This was certainly reflected in a significant increase in the number of primordial follicles. It has been shown that PCNA is also tangled in DNA repair. Thus, DNA polymerase delta might be activated to repair possible damage to the genetic material in the oocytes selected to grow [77]. Increased PCNA expression in the oocyte nucleus could also reflect the increased expression of growth factors in the oocyte, as PCNA expression can be stimulated by various growth factors even in quiescent cells.

Taken together, the results of this study suggest the presence of potential VSELs in rat ovaries that they survive chemotherapy, are modulated by FSH and enhanced by a combination of FSH and MSC, and retain the ability to undergo oocyte-specific differentiation.

## Conclusion

MSC co-administration improved the local microenvironment of the ovary and integrated with PMSG to promote follicular development into mature oocytes apparently by granulosa cell function. The accompanying evidence of postnatal oogenesis provides relevance to women who experience premature ovarian failure because of oncotherapy.

## Data Availability

All data generated and/or analyzed during this study are included in this published article.

## Abbreviations

PF: Primordial follicles
FSH: Follicle-stimulating hormone
PMSG: Pregnant mare serum gonadotropin
BM-MSCs: Bone marrow-derived mesenchymal stem cells
OF: Ovarian failure
PSCs: Pluripotent stem cells
GSCs: Germline stem cells
OSE: Ovarian surface epithelium
VSELs: Very small embryonic-like stem cells
OGSCs: Ovary germ stem cells
OCT-4: Octamer-binding transforming factor 4
PCNA: Proliferating cell nuclear antigen
SCP3: Synaptonemal complex protein 3
Mvh: Mouse VASA homolog
Nobox: NOBOX oogenesis homeobox
DAZL: Deleted in azoospermia like
Nanog: Nanog Homeobox
Sca-1: Stem cell antigen-1
FSHr: Follicle-stimulating hormone receptor
Oct-4A: Octamer-binding transcription factor 4 isoform A
Stra8: Stimulated by retinoic acid 8
BAX: Bcl-2-associated X protein
GAPDH: Glyceraldehyde-3-phosphate dehydrogenase
